# A synthetic peptide targeting the BH4 domain of Bcl-2 induces apoptosis in multiple myeloma and follicular lymphoma cells alone or in combination with agents targeting the BH3-binding pocket of Bcl-2

**DOI:** 10.18632/oncotarget.4489

**Published:** 2015-07-11

**Authors:** Andrew R. Lavik, Fei Zhong, Ming-Jin Chang, Edward Greenberg, Yuvraj Choudhary, Mitchell R. Smith, Karen S. McColl, John Pink, Frederic J. Reu, Shigemi Matsuyama, Clark W. Distelhorst

**Affiliations:** ^1^ Division of Hematology/Oncology, Case Western Reserve University School of Medicine and University Hospitals Case Medical Center, Cleveland, Ohio, USA; ^2^ Department of Medicine, MetroHealth Medical Center and Case Western Reserve University School of Medicine, Cleveland, Ohio, USA; ^3^ Department of Translational Hematology and Oncology Research, Taussig Cancer Institute, The Cleveland Clinic Foundation, Cleveland, Ohio, USA; ^4^ Case Comprehensive Cancer Center, Cleveland, Ohio, USA

**Keywords:** Bcl-2, inositol 1,4,5-trisphosphate receptor, ABT-199, Bruton’s tyrosine kinase, lymphoid malignancy

## Abstract

Bcl-2 inhibits apoptosis by two distinct mechanisms but only one is targeted to treat Bcl-2-positive malignancies. In this mechanism, the BH1-3 domains of Bcl-2 form a hydrophobic pocket, binding and inhibiting pro-apoptotic proteins, including Bim. In the other mechanism, the BH4 domain mediates interaction of Bcl-2 with inositol 1,4, 5-trisphosphate receptors (IP_3_Rs), inhibiting pro-apoptotic Ca^2+^ signals. The current anti-Bcl-2 agents, ABT-263 (Navitoclax) and ABT-199 (Venetoclax), induce apoptosis by displacing pro-apoptotic proteins from the hydrophobic pocket, but do not inhibit Bcl-2-IP_3_R interaction. Therefore, to target this interaction we developed BIRD-2 (Bcl-2 IP_3_ Receptor Disruptor-2), a decoy peptide that binds to the BH4 domain, blocking Bcl-2-IP_3_R interaction and thus inducing Ca^2+^-mediated apoptosis in chronic lymphocytic leukemia, multiple myeloma, and follicular lymphoma cells, including cells resistant to ABT-263, ABT-199, or the Bruton’s tyrosine kinase inhibitor Ibrutinib. Moreover, combining BIRD-2 with ABT-263 or ABT-199 enhances apoptosis induction compared to single agent treatment. Overall, these findings provide strong rationale for developing novel therapeutic agents that mimic the action of BIRD-2 in targeting the BH4 domain of Bcl-2 and disrupting Bcl-2-IP_3_R interaction.

## INTRODUCTION

The Bcl-2 protein is elevated in a variety of types of cancer, including those derived by malignant transformation of B-lymphocytes: chronic lymphocytic leukemia (CLL), follicular lymphoma (FL), and multiple myeloma (MM) [1]. Bcl-2 elevation contributes to apoptosis resistance in malignant cells, a hallmark of cancer that represents a major hurdle in cancer treatment [2]. Bcl-2 is the founding member of a large family of apoptosis regulating proteins composed of one or more Bcl-2 homology (BH) domains [3, 4]. Bcl-2 has four BH domains (BH1-4), typical of most anti-apoptotic family members, while pro-apoptotic family members lack the BH4 domain and have only BH1-3 domains (*e.g*., Bax, Bak). Another subset of pro-apoptotic family members has only a BH3 domain (*e.g*., Bim). These BH3-only proteins act as sentinels of cell stress that activate Bax or Bak, which in turn trigger apoptosis by permeabilizing the outer mitochondrial membrane and releasing cytochrome *c*. Bcl-2 binds these pro-apoptotic BH3-only family members, thereby inhibiting apoptosis.

A major advance in cancer treatment is the development of the BH3 mimetic agents ABT-737, its orally bioavailable analog ABT-263 (Navitoclax), and ABT-199 (Venetoclax) [5, 6]. These small molecules competitively inhibit BH3-only protein binding by Bcl-2, inducing apoptosis in cells addicted to Bcl-2 elevation for survival [7]. The most recent, ABT-199, selectively targets Bcl-2 and shows considerable promise for treatment of a wide range of Bcl-2-positive malignancies [6–9].

But interaction of Bcl-2 with BH3-only proteins is not the only way Bcl-2 represses apoptosis. Bcl-2 also binds to the inositol 1,4,5-trisphosphate receptors (IP_3_Rs), Ca^2+^ channels located on the endoplasmic reticulum (ER), preventing high amplitude intracellular Ca^2+^ elevations that trigger cell death [10, 11]. Bcl-2 accomplishes this by docking the Ca^2+^-activated protein phosphatase calcineurin and the calcineurin-regulated inhibitor of protein phosphatase 1, DARPP-32, to IP_3_Rs. This complex forms a negative feedback loop that senses excessive Ca^2+^ release and rapidly decreases IP_3_R phosphorylation, thereby decreasing IP_3_R-mediated Ca^2+^ release and thus preventing apoptosis [12].

Distinct regions of the Bcl-2 protein govern these disparate mechanisms of apoptosis regulation. BH domains 1-3 participate in forming a hydrophobic pocket in which pro-apoptotic proteins are bound [3]. The BH3 mimetic agents induce apoptosis by binding in this pocket and displacing pro-apoptotic proteins [4]. On the other hand, the BH4 domain of Bcl-2 mediates the binding of Bcl-2 to IP_3_Rs [13, 14].

Currently there is no therapeutic agent designed to target the BH4 domain of Bcl-2. Therefore, to selectively target Bcl-2-IP_3_R interaction, we developed a synthetic peptide corresponding to the IP_3_R binding site for Bcl-2 [15]. This peptide disrupts Bcl-2-IP_3_R interaction, reversing Bcl-2′s inhibition of IP_3_-induced Ca^2+^ elevation and decreasing cell survival, without disturbing the binding of pro-apoptotic proteins by Bcl-2 [13, 15]. Elimination of a potential protease cleavage site produced a more potent version of this peptide capable of inducing Ca^2+^-mediated apoptosis in primary human CLL cells (but not in normal human lymphocytes) [16] and in diffuse large B-cell lymphoma cell lines [17]. Here we call this peptide BIRD-2 (Bcl-2-IP_3_R Disruptor-2). BIRD-2 binds directly to the BH4 domain of Bcl-2, functioning as a decoy peptide to competitively inhibit Bcl-2 interaction with IP_3_Rs, reversing Bcl-2′s control over IP_3_R-mediated Ca^2+^ release and thereby inducing high amplitude intracellular Ca^2+^ elevations that trigger apoptosis [14, 16].

Knowledge that Bcl-2 inhibits apoptosis by two distinct validated pathways involving separate regions of the Bcl-2 protein suggests that targeting the BH4 domain of Bcl-2 should be undertaken as a novel therapeutic strategy. But whether or not this approach might represent a potential therapeutic gain in view of the already established approach to targeting Bcl-2 with BH3 mimetic agents has not been tested. Therefore, the present work was undertaken in which we employ a series of human myeloma cell lines (HMCLs) to compare responses to BIRD-2 and ABT-263/ABT-199. The findings indicate for the first time that BIRD-2 induces apoptosis both *in vitro* and *in vivo*. Also, we find that treatment of HMCLs with BIRD-2 together with ABT-263/ABT-199 enhances cell killing. This combined activity extends to FL cells as well. Moreover, we demonstrate that BIRD-2 induces apoptosis in HMCLs resistant to both ABT-263/ABT-199 and to the Bruton’s tyrosine kinase (Btk) inhibitor Ibrutinib. Overall, the findings of the present report provide strong rationale for developing novel therapeutic agents that mimic the action of BIRD-2 in targeting the BH4 domain of Bcl-2.

## RESULTS

### BIRD-2 activity *in vitro* and *in vivo* in HMCLs

**Figure 1:**
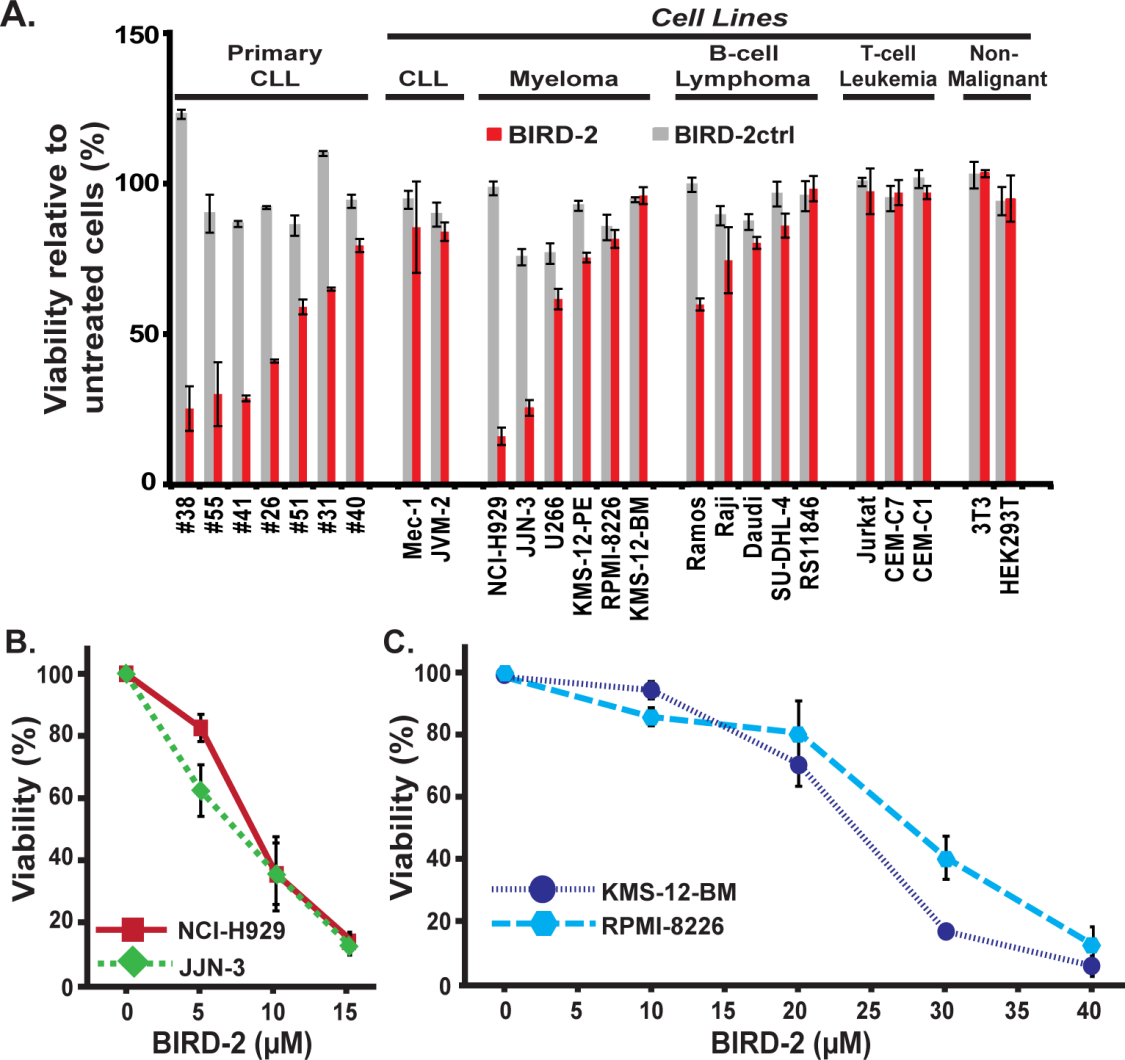
Differential sensitivity of various malignant and non-malignant cell lines to BIRD-2-induced cell death **A.** Primary human CLL cells, cell lines representing a variety of lymphoid malignancies, and non-lymphoid cell lines were treated with 10 μM BIRD-2 or BIRD-2ctrl for 24 hr and viability was assessed using an MTS assay. Findings represent mean ± SD from three independent experiments. **B.** and **C.** HMCLs were incubated with BIRD-2 at multiple dose levels for 24 hr and viability assessed using the MTS assay. BIRD-2ctrl did not induce cell death at the same dose levels (not shown). Symbols represent mean ± SD in at least two separate experiments performed in triplicate.

An MTS assay was used to compare the BIRD-2 sensitivity of primary human CLL cells with cell lines representing various other lymphoid malignancies and transformed lines of non-malignant derivation in relation to sensitivity to BIRD-2ctrl, a control peptide derived from BIRD-2 sequence. The MTS assay determines NAD(P)H-dependent cellular oxidoreductase activity as a measure of cell viability. Cell lines corresponding to B-cell lymphoid malignancies, including MM and B-cell lymphoma, were generally more sensitive to BIRD-2 than lines corresponding to T-cell malignancies and non-malignant cell lines (Figure 1A). The six HMCLs demonstrate a range of sensitivities to BIRD-2, as observed with primary human CLL cells (Figure 1A). Dose responses for the two most sensitive HMCLs, NCI-H929 and JJN-3, and the two least sensitive HMCLs, KMS-12-BM and RPMI-8226, are shown in Figure 1B and Figure 1C, respectively.

**Figure 2:**
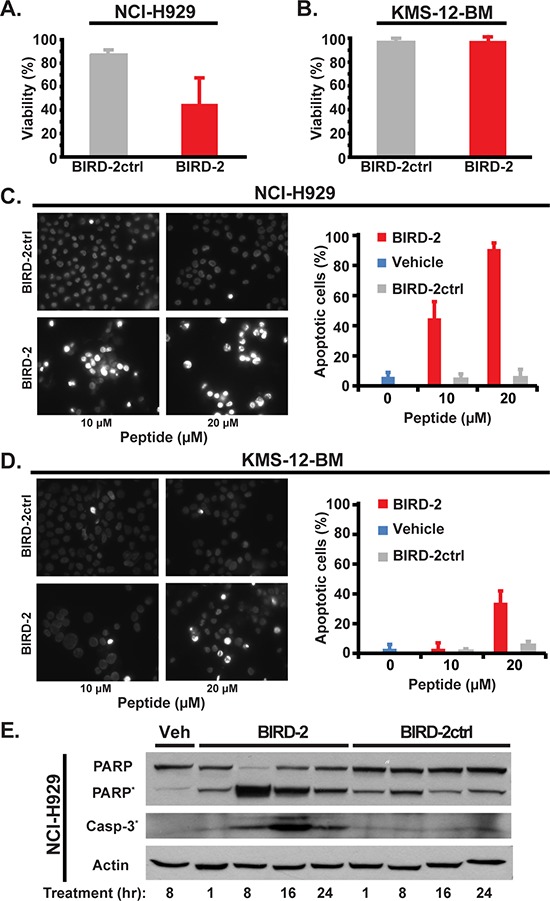
Apoptosis induction by BIRD-2 **A.** and **B.** NCI-H929 (A) and KMS-12-BM (B) cell viability by MTS assay 24 hr after treatment with 10 μM BIRD-2 *vs* BIRD-2ctrl (mean ± SD of at least 3 experiments). **C.** and **D.** *Left*: representative epifluorescence images of Hoechst 33342-stained NCI-H929 (C) and KMS-12-BM (D) cells treated with 10 or 20 μM BIRD-2 or BIRD-2ctrl for 24 hr. Bright staining of nuclei is due to nuclear condensation and is characteristic of apoptosis, while dim staining indicates live cells. *Right*: quantification of apoptotic Hoechst 33342-stained nuclei. Data expressed as mean ± SD of over 200 cells per treatment group. **E.** Immunoblotting displaying relative levels of PARP, cleaved PARP*, and cleaved Caspase-3* in NCI-H929 cells treated with either vehicle (Veh) water, 10 μM BIRD-2 or BIRD-2ctrl for the indicated times. Beta actin is loading control.

**Figure 3:**
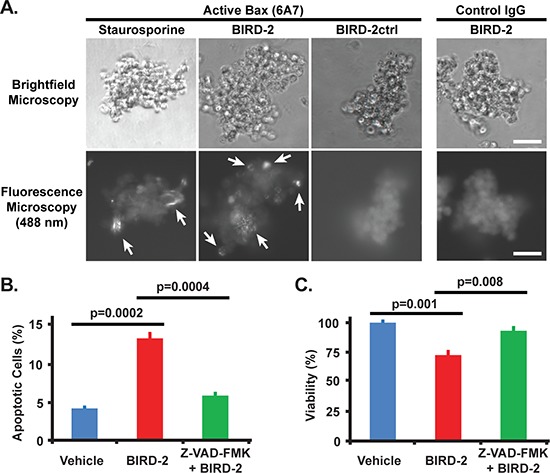
Bax activation by BIRD-2 and protection from BIRD-2-induced cell death by caspase inhibition **A.** Representative immunocytochemistry images demonstrating the activation and oligomerization of Bax in NCI-H929 cells 4 hr post treatment with 10 μM BIRD-2, 10 μM BIRD-2ctrl, or 2.5 μM staurosporine. Cells were stained with the Bax 6A7 antibody, which specifically detects the active form of Bax, or with normal mouse IgG (control), and were imaged by brightfield (*top row*) or fluorescence (*bottom row*) microscopy. Arrows indicate active oligomerized Bax. Scale bar, 50 μm. **B.** Quantification of apoptotic Hoechst 33342-stained nuclei in NCI-H929 cells after 1 hr pretreatment with 200 μM Z-VAD-FMK or DMSO (vehicle) followed by 4 hr treatment with 10 μM BIRD-2. Data expressed as mean ± SEM of three quantifications of over 200 cells per treatment group. **C.** CellTiter-Glo viability assay in NCI-H929 cells pretreated for 1 hr with 200 μM Z-VAD-FMK or DMSO followed by 4 hr treatment with 10 μM BIRD-2. Data expressed as mean ± SEM of triplicate measurements.

The differential sensitivity of NCI-H929 and KMS-12-BM cells to 10 μM BIRD-2 treatment for 24 hours, as measured by MTS assay (Figure 2A, 2B), is substantiated by quantifying the percentage of apoptotic nuclei developing after BIRD-2 treatment (Figure 2C, 2D). The BIRD-2-mediated decline in survival is accompanied by a larger dose-dependent increase in the percentage of NCI-H929 cells exhibiting apoptotic nuclear chromatin condensation than observed in KMS-12-BM cells. The findings indicate that KMS-12-BM cells are not completely resistant to BIRD-2, but require a much higher concentration of BIRD-2 than NCI-H929 cells to achieve a similar degree of apoptosis. In addition, we find that BIRD-2 treatment also induces PARP cleavage and caspase-3 activation in NCI-H929 cells, providing further evidence of apoptosis induction by BIRD-2 (Figure 2E). Consistent with these findings, treatment of NCI-H929 cells with BIRD-2 causes activation and oligomerization of the mitochondrial membrane-localized pro-apoptotic protein Bax, which permeabilizes the mitochondrial outer membrane during apoptosis initiation. This finding is evident in cells treated with BIRD-2 or the positive control staurosporine by the formation of perinuclear puncta detected with the Bax 6A7 antibody, which specifically recognizes the conformationally active form of Bax (Figure 3A). Moreover, pretreatment with the caspase inhibitor Z-VAD-FMK protects NCI-H929 cells from BIRD-2-induced formation of apoptotic nuclei (Figure 3B) and cell death measured by the CellTiter-Glo (CTG) assay, which quantifies ATP levels as a measure of cell viability (Figure 3C). These findings indicate that the cell death induced by BIRD-2 is primarily caspase-dependent.

**Figure 4:**
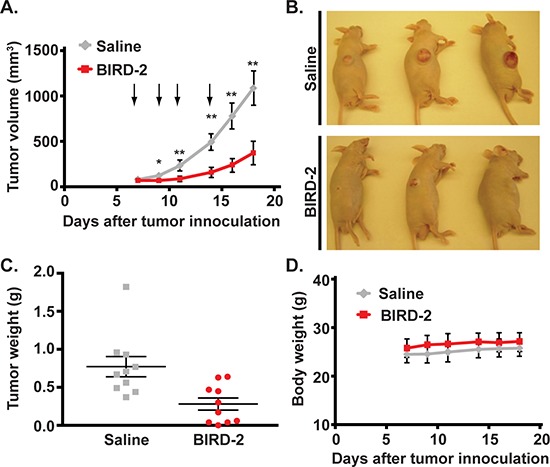
BIRD-2 induces HMCL cell death *in vivo* NCI-H929 cells (9 × 10^6^) were injected subcutaneously into the flank region of NCR nu/nu mice. When tumor volume reached an average of 60–80 mm^3^, mice were injected at the site of tumors with 20 μL saline or BIRD-2 at a dose of 10 mg per kg body weight. **A.** Growth of xenograft tumors in mice treated with either saline or BIRD-2. Arrows indicate time of treatment. Data points indicate mean ± SEM of three independent experiments with at least three mice per treatment group; *, *p* < 0.05; **, *p* < 0.01. **B.** Representative saline- and BIRD-2-treated mice imaged at the completion of an experiment. **C.** Weight of tumors excised from saline-treated or BIRD-2-treated mice. Data are expressed as mean ± SEM. **D.** Body weight of mice treated with either saline or BIRD-2. *p* > 0.05 for all data points.

To examine the anti-tumor effect of BIRD-2 on HMCLs *in vivo*, NCI-H929 cells were inoculated subcutaneously into the flank regions of nude mice. Once tumors had become visible, tumor-bearing mice were injected with either BIRD-2 or saline at the site of tumors. Administration of 10 mg/kg BIRD-2 every 2-3 days for a total of four injections caused a significant reduction in tumor growth rate compared to the control group (Figure 4A, 4B). Furthermore, injection of BIRD-2 inhibited end-point tumor weight to less than 50% compared to the control group (Figure 4C). Notably, there was not a significant difference in body weight between the two treatment groups, suggesting that BIRD-2 injection does not cause apparent toxicity in mice (Figure 4D). Together, these findings indicate that percutaneous injection of BIRD-2 penetrates tissue through diffusion and inhibits tumor growth without inducing immediate recognizable toxicity in mice and provide evidence of *in vivo* single-agent activity of targeting Bcl-2′s BH4 domain.

### Reciprocal sensitivity of HMCLs to BIRD-2 and BH3 mimetic agents

**Figure 5:**
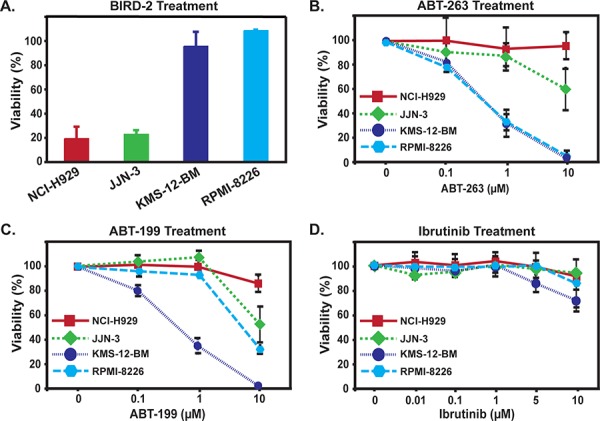
Reciprocal sensitivity of HMCLs to BIRD-2 and ABT-263, ABT-199, or Ibrutinib **A.** Differential sensitivity of HMCLs to treatment with 10 μM BIRD-2 for 24 hr measured by CTG viability assay, consistent with preceding findings (Figure 1) using MTS assay. **B.** HMCLs were treated for 24 hr with a range of concentrations of ABT-263. Findings indicate that KMS-12-BM and RPMI-8226 cells are much more sensitive to ABT-263 than are NCI-H929 or JJN-3 cells. **C.** HMCLs were treated for 24 hr with a range of concentrations of ABT-199. Findings indicate that KMS-12-BM cells are much more sensitive to ABT-199 than are NCI-H929, JJN-3 and RPMI-8226 cells. In panels A–C, cell viability was measured by CellTiter-Glo Assay, performed in triplicate in at least three experiments and displayed as mean ± SD. **D.** HMCLs were incubated with Ibrutinib at multiple dose levels for 24 hr and viability was assessed by CellTiter-Glo assay. Symbols represent mean ± SD of triplicate measurements; a representative experiment is shown.

The sensitivities of HMCLs to BIRD-2 and ABT-263 or ABT-199 were compared using the CTG assay (Figure 5). The marked difference in BIRD-2 sensitivity of NCI-H929 and JJN-3 *versus* KMS-12-BM and RPMI-8226 cells using the MTS assay in Figure 1 and the apoptotic nuclear chromatin condensation assay in Figure 2 was confirmed using the CTG assay (Figure 5A). ABT-263 induced an appreciable degree of cell death in only two of the HMCLs, KMS-12-BM and RPMI-8226, while ABT-199 induced a comparable degree of cell death only in KMS-12-BM cells (Figure 5B, 5C). By comparison, all HMCLs exhibited considerable resistance to the Btk inhibitor Ibrutinib (Figure 5D). These findings indicate that BIRD-2 is capable of inducing death in HMCLs that are resistant to both the BH3 mimetic agents ABT-263 and ABT-199 and to the Btk inhibitor Ibrutinib. Moreover, the findings suggest a reciprocal sensitivity of HMCLs to BIRD-2 *versus* ABT-263/199, consistent with the different mechanisms of action of these agents.

**Figure 6:**
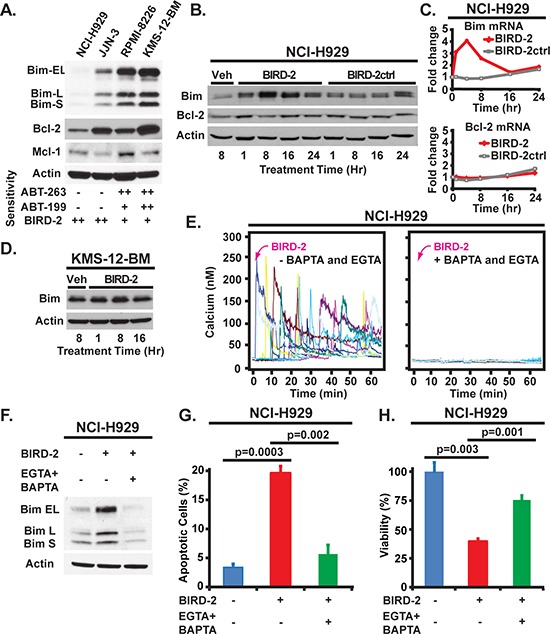
Bim levels and their regulation by BIRD-2 in HMCLs **A.** Immunoblot showing Bim, Bcl-2, and Mcl-1 levels in HMCLs. The sensitivity of cell lines to BH3 mimetic agents and to BIRD-2 is summarized at bottom. **B.** Immunoblot showing relative levels of Bim (long form) and Bcl-2 in NCI-H929 cells treated with either Veh, 10 μM BIRD-2 or BIRD-2ctrl for the indicated times. **C.** Relative Bim and Bcl-2 mRNA levels in NCI-H929 cells treated with 10 μM BIRD-2 or BIRD-2ctrl for times shown. Beta-actin mRNA levels are used in data normalization. Data are presented as mean ± SD of triplicate samples from a representative of two independent experiments. **D.** Immunoblot showing levels of Bim (long form) in KMS-12-BM cells treated with either Veh or BIRD-2 for the indicated times. **E.** Representative Ca^2+^ traces by single cell digital imaging documenting Ca^2+^ elevation induced by BIRD-2 (*left panel*) and its inhibition by EGTA + BAPTA (*right panel*). **F.** Immunoblot showing Bim elevation in NCI-H929 cells induced by BIRD-2 treatment for 4 hr. EGTA (10 mM) and BAPTA-AM (1 μM) were added to cells 30 min before BIRD-2 addition to chelate Ca^2+^. Beta-actin is loading control in each of the immunoblots. **G.** and **H.** Hoechst 33342 staining for apoptotic nuclei (G) and CTG viability assay (H) of NCI-H929 cells pre-treated for 30 min with EGTA (10 mM) and BAPTA-AM (1 μM) to chelate Ca^2+^ followed by 4 hr treatment with 10 μM BIRD-2. Data represent mean ± SEM of triplicate measurements.

The Bcl-2 family protein Bim is a key mediator of apoptosis in lymphoid malignancies [18, 19]. As a BH3-only protein, Bim levels contribute to differential sensitivity of cells to BH3 mimetic agents by priming Bcl-2-positive cells for death [20]. Therefore, in order to explore differences in sensitivity of the HMCLs to BH3 mimetic agents, we compared expression levels of Bim in these cells. We find that Bim protein is expressed highly in the BH3 mimetic-sensitive HMCLs, whereas Bim expression is low in the BH3 mimetic agent-resistant NCI-H929 and JJN-3 lines (Figure 6A). By comparison, Bcl-2 and Mcl-1 are expressed at varying levels (Figure 6A).

Moreover, we observe that in highly BIRD-2-sensitive NCI-H929 cells, BIRD-2 induces elevation of Bim protein and mRNA, while Bcl-2 mRNA and protein levels are essentially unchanged (Figure 6B, 6C). On the other hand, in KMS-12-BM cells, basal Bim levels are already high and change little following BIRD-2 treatment (Figure 6D). As shown in earlier work, disruption of Bcl-2-IP_3_R interaction by BIRD-2 induces IP_3_R-mediated Ca^2+^ elevation [12, 15–17] (Figure 6E, *left*). Adding the Ca^2+^ chelators EGTA and BAPTA-AM to cells prior to BIRD-2 treatment inhibits BIRD-2-mediated Ca^2+^ elevation (Figure 6E, *right*) and Bim elevation (Figure 6F), and prevents BIRD-2-induced formation of apoptotic nuclei and loss of cell viability (Figure 6G, 6H). Therefore, BIRD-2-induced Bim elevation in NCI-H929 cells is Ca^2+^-mediated, consistent with earlier evidence of a role for Ca^2+^-induced transcriptional elevation of Bim in lymphocyte cell death [21]. Moreover, these findings demonstrate that the cell death induced by BIRD-2 is largely Ca^2+^-dependent.

### Combining BIRD-2 with BH3 mimetic agents

**Figure 7:**
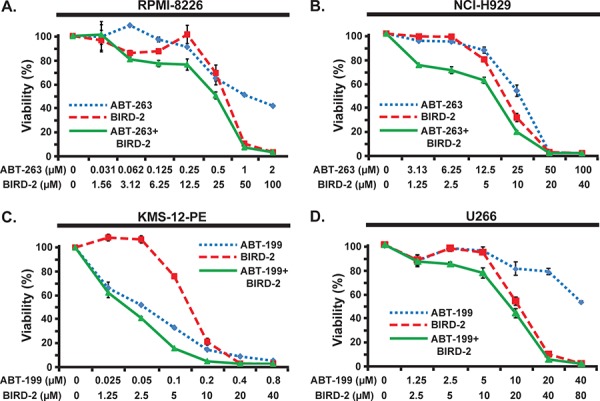
Combined activity of BIRD-2 and BH3 mimetic agents **A.** and **B.** RPMI-8226 (A) or NCI-H929 (B) cells were treated with the indicated concentrations of ABT-263, BIRD-2, or ABT-263 and BIRD-2 in combination. Cell viability was measured after 24 hr by CellTiter-Glo assay. Results are mean ± SEM of triplicate measurements. **C.** and **D.** KMS-12-PE (C) or U266 (D) cells were treated with the indicated concentrations of ABT-199, BIRD-2, or ABT-199 and BIRD-2 in combination. Cell viability was measured after 24 hr by CellTiter-Glo assay. Results are mean ± SEM of triplicate measurements.

The observation that BIRD-2 treatment elevates Bim mRNA and protein without elevating Bcl-2 raises the possibility that BIRD-2 may sensitize HMCLs to BH3 mimetic agents by increasing the ratio of Bim to Bcl-2. Therefore, we treated HMCLs with BIRD-2 in conjunction with BH3 mimetic agents. In ABT-263-sensitive RPMI-8226 cells, dual treatment with BIRD-2 and ABT-263 enhanced cell death induction compared to either agent alone (Figure 7A). Moreover, co-treatment with BIRD-2 and ABT-263 also enhanced cell death induction in ABT-263/ABT-199-resistant cells as shown for NCI-H929 cells in Figure 7B. This effect was observed most notably at concentrations of BIRD-2 and ABT-263 that caused little single agent cell death (Figure 7B). To test the potential of combining BIRD-2 with ABT-199, highly ABT-199-sensitive KMS-12-PE cells (ABT-199 EC_50_ 60 nM) and highly ABT-199-resistant U266 cells (ABT-199 EC_50_ > 40 μM) were treated with both agents. Combined treatment with BIRD-2 and ABT-199 augmented cell death induction in both KMS-12-PE and U266 HMCLs compared to single agent treatment (Figure 7C, 7D). These findings indicate that dual treatment with BIRD-2 and ABT-263 or ABT-199 enhances cell death induction in a range of HMCLs exhibiting both sensitivity and resistance to BH3 mimetic agents.

**Figure 8:**
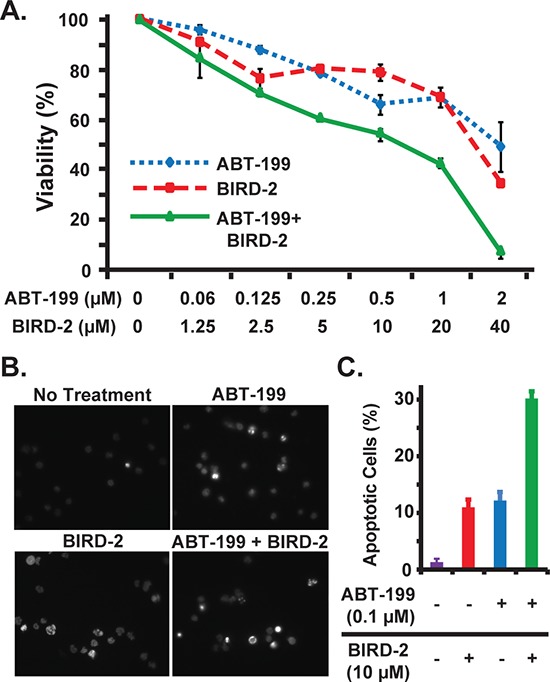
Combined activity of BIRD-2 and ABT-199 in follicular lymphoma cells **A.** WSU-FSCCL follicular lymphoma cells were treated with the indicated concentrations of ABT-199, BIRD-2, or ABT-199 and BIRD-2 in combination. Cell viability was measured after 24 hr by CellTiter-Glo assay. Results are mean ± SEM of triplicate measurements. **B.** Representative epifluorescence images of Hoechst 33342-stained WSU-FSCCL cells treated with 0.1 μM ABT-199 and 10 μM BIRD-2 either alone or in combination for 20 hr. Bright staining of nuclei is due to nuclear condensation and is characteristic of apoptosis, while dim staining indicates live cells. **C.** Quantification of apoptotic Hoechst 33342-stained nuclei. Data expressed as mean ± SEM of over 200 cells per treatment group.

A similar approach was taken in the WSU-FSCCL human FL cell line, which exhibits the characteristic t(14;18) chromosomal translocation responsible for elevated Bcl-2 expression in FL [22] (Figure 8). As in HMCLs, cytotoxicity (Figure 8A) and apoptosis (Figure 8B, 8C) are increased when BIRD-2 and ABT-199 are combined, providing evidence that BIRD-2 functions together with BH3 mimetic agents in other Bcl-2-positive malignancies in addition to MM.

## DISCUSSION

**Figure 9:**
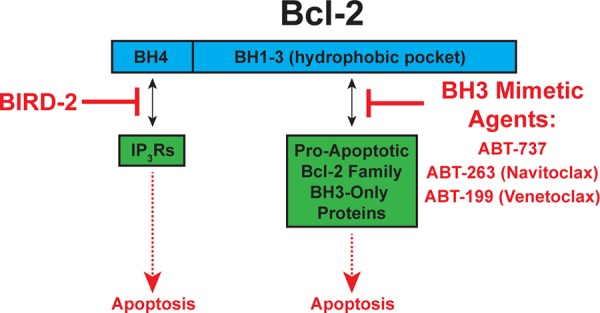
Targeting Bcl-2′s two anti-apoptotic mechanisms *Left*: Bcl-2 binds IP_3_Rs and prevents Ca^2+^ elevation capable of inducing apoptosis. BIRD-2 inhibits Bcl-2-IP_3_R interaction, thereby inducing apoptosis. *Right*: Bcl-2 binds BH3-only proteins and prevents them from inducing apoptosis. BH3 mimetic agents, such as ABT-263 and ABT-199, release BH3-only proteins from Bcl-2, thereby inducing apoptosis.

Bcl-2 inhibits apoptosis by two disparate mechanisms [10, 11] (Figure 9). On the one hand, Bcl-2 binds BH3-only pro-apoptotic proteins and prevents them from inducing apoptosis; BH3 mimetic agents, such as ABT-263 and ABT-199, inhibit this mechanism. On the other hand, Bcl-2 binds IP_3_Rs and prevents Ca^2+^ elevation capable of inducing apoptosis (Figure 9). This mechanism and its inhibition by BIRD-2 are summarized elsewhere [10]. Here we extend our earlier work indicating that BIRD-2 induces apoptosis in primary human CLL cells [16] by showing for the first time that BIRD-2 also induces apoptosis in MM and FL cells. Moreover, we demonstrate that BIRD-2 treatment induces Bax activation and cell death that is in large part Ca^2+^- and caspase-dependent. Perhaps even more importantly, the present work indicates that BIRD-2 induces apoptosis in MM cells insensitive to ABT-263, ABT-199, and/or Ibrutinib.

The resistance of certain HMCLs to ABT-263 and ABT-199 in the present study is not surprising, as work by other investigators indicates that only a subset of HMCLs are sensitive to BH3 mimetic agents [23, 24]. The observation that BIRD-2 kills BH3 mimetic agent-resistant HMCLs is fully consistent with the dual mechanisms of Bcl-2 action illustrated in Figure 9 and provides strong rationale for developing therapeutic agents that mimic the action of BIRD-2. Moreover, these findings indicate that two separate types of addiction to Bcl-2 exist among malignant cells: one dependent on Bcl-2′s BH1-3 domain for binding and sequestering of pro-apoptotic BH3-only proteins (vulnerable to BH3 mimetic agents) [7, 8], and the other dependent on Bcl-2′s BH4 domain for binding IP_3_Rs and inhibiting pro-apoptotic Ca^2+^ elevations (vulnerable to BIRD-2). Therefore, fully understanding and targeting the anti-apoptotic Bcl-2-IP_3_R interaction in addition to Bcl-2-BH3-only protein interaction should enable more complete interference with Bcl-2, a critical defense mechanism of malignant cells against apoptosis.

Because Bcl-2′s dual anti-apoptotic mechanisms are so different from each other, it theoretically should be possible to achieve a better therapeutic action by combining agents targeting each of the mechanisms. Indeed, the findings of the present work suggest that combinations of BIRD-2 and either ABT-263 or ABT-199 induce more cell death than either agent alone, in both HMCLs and in t(14;18)-positive FL cells. Moreover, the observation that BIRD-2 treatment elevates Bim without elevating Bcl-2 suggests that BIRD-2 may sensitize cells to BH3 mimetic agents. The BH3-only protein Bim is already known to play an important role in mediating apoptosis of malignant lymphocytes [20, 25–28]. Furthermore, when basal Bim levels are elevated, malignant cells can become addicted to Bcl-2 for its ability to bind Bim and prevent it from inducing apoptosis [7, 8]. BH3 mimetic agents induce apoptosis by displacing Bim from Bcl-2 [7, 8]. Cells with high levels of both Bcl-2 and Bim are primed for cell death induction by BH3 mimetic agents. Consistent with this, HMCLs found in the present work to be killed by ABT-263 or ABT-199 have high levels of Bim, whereas the HMCLs resistant to BH3 mimetic agents have low levels of Bim. Combining BIRD-2 with ABT-263 or ABT-199 may be particularly advantageous, we propose, since BIRD-2 treatment elevates cytoplasmic Ca^2+^, which in turn elevates Bim levels. In short, certain HMCLs are primed to respond to BH3 mimetic agents because they contain a high concentration of BH3-only proteins like Bim that are sequestered by highly expressed Bcl-2. In HMCLs with relatively low basal levels of Bim, there are too few BH3-only proteins to be released from Bcl-2 and mediate cell death in response to BH3 mimetic agents unless they are also treated with BIRD-2, which elevates Bim levels, thus priming the cells for sensitivity to BH3 mimetic agents.

By interacting with IP_3_Rs, we posit that Bcl-2 regulates a key pro-survival signaling pathway in many B-cell malignancies, driven by elevated expression and/or activity of Btk. Btk phosphorylates and thereby activates phospholipase *C* gamma (PLCγ), leading to protein kinase *C* (PKC) activation through the generation of diacylglycerol (DAG) [29]. Novel therapeutic agents that inhibit Btk, such as Ibrutinib, inhibit survival pathways including PI3K/AKT and the PKC-mediated MAPK and NFκB pathways, decreasing the proliferation and survival of B-cell malignancies [30–32]. Ibrutinib has recently been approved for use in B-cell malignancies CLL and mantle cell lymphoma, is currently in phase II clinical trials for use in MM [33, 34], and is rapidly becoming one of the most effective targeted agents for B-cell malignancies [35]. However, Ibrutinib resistance is emerging [36, 37], which results most often from Ibrutinib binding site mutations in Btk or gain-of-function mutations in PLCγ [38–40], both of which lead to increased signaling flux through PLCγ. Importantly, since PLCγ produces IP_3_ concurrently with DAG, a consequence of Ibrutinib resistance is likely to be increased IP_3_ generation, with the expected result being increased IP_3_R-dependent Ca^2+^ elevation, were it not for the inhibitory presence of Bcl-2 on IP_3_Rs. Therefore, we anticipate that BIRD-2 might overcome Ibrutinib resistance by displacing Bcl-2 from IP_3_Rs and inducing Ca^2+^ elevation and apoptosis downstream of hyperactive PLCγ. Although this hypothesis has not been formally tested, our observation that BIRD-2 robustly induces apoptosis in NCI-H929 and JJN-3 cells, two HMCLs resistant to Ibrutinib, is consistent with the concept that BIRD-2 may be effective in some situations where Ibrutinib is not. These findings are intriguing since both Btk and Bcl-2 are highly expressed in B-cell malignancies, including MM [1, 33, 35–41].

Similar to our findings using BIRD-2 in combination with ABT-263/ABT-199, we observed that co-treatment of HMCLs with BIRD-2 and Ibrutinib enhanced cell killing, most notably when treated continuously with Ibrutinib at concentrations greater than 1 μM (data not shown). We additionally tested a more stringent Ibrutinib dosing schedule comprised of short-term treatment (2 hours) with low dose Ibrutinib (1 μM) followed by Ibrutinib washout and addition of BIRD-2. Under these conditions, there was no enhancement of cell killing by dual treatment with Ibrutinib and BIRD-2 compared to BIRD-2 treatment alone (data not shown). Given that Ibrutinib potently and irreversibly inhibits Btk at concentrations well below 1 μM [42] and that clinical Ibrutinib dosing schedules in patients result in full occupancy of the Btk binding site by Ibrutinib for at least 24 hours post-treatment [43], the more stringent Ibrutinib dosing schedule would be expected to completely inhibit Btk throughout the experiment period at a dose of 1 μM. Taken together, these results suggest that the enhancement of cell killing observed following co-treatment of HMCLs with BIRD-2 and Ibrutinib at Ibrutinib concentrations above 1 μM may be due to effects of Ibrutinib on targets other than Btk. Intriguingly, Ibrutinib is known to inhibit a variety of kinases in addition to Btk [42, 44, 45], indicating that BIRD-2 may be effective in combination with inhibitors of kinases other than Btk.

We initially employed HMCLs in the present work as a tool for investigating Bcl-2′s dual anti-apoptotic mechanisms and the effects of targeting these mechanisms with either BIRD-2 or BH3 mimetic agents alone, or in combination. At the same time, our findings provide rationale for treating MM and also FL with agents that target Bcl-2-IP_3_R interaction, alone or in combination with BH3 mimetic agents. Success in this regard should have a major impact, since both MM and FL remain challenging, incurable malignancies despite a number of recent therapeutic advances [46–48]. Bcl-2 is expressed in MM cells at levels comparable to FL, even though MM cells lack the t(14;18) translocation responsible for elevating Bcl-2 in FL cells [41]. In some but not all HMCLs and primary patient samples the BH3 mimetic ABT-737 induces apoptosis as a single agent and also demonstrates synergistic activity with various chemotherapeutic agents [49–51]. Recent studies also indicate responsiveness of a small subset of HMCLs and primary patient samples to the Bcl-2 selective BH3 mimetic agent ABT-199 [23]. However, ABT resistance is present in more than half of primary MM samples treated with ABT-737 and ABT-199 [23, 52]. Here we find that BIRD-2 induces apoptosis in HMCLs resistant to BH3 mimetic agents ABT-263 and ABT-199 and to the Btk inhibitor Ibrutinib; moreover, one of the BIRD-2-sensitive lines, NCI-H929, corresponds to a MM subgroup that is not only resistant to BH3 mimetic agents but also belongs to a poor prognosis MM category associated with a t(4;14) chromosomal translocation [24]. Therefore, the findings of the present study offer hope that developing therapeutic agents that mimic the action of BIRD-2 will have a major effect in treating MM, either as single agents or in combination with BH3 mimetic agents.

Since discovering that Bcl-2 interacts with IP_3_Rs to regulate IP_3_-mediated Ca^2+^ elevation in lymphoma cells [53], our intention has been to target this interaction for cancer treatment. Broad interest in the role of IP_3_Rs and Ca^2+^ signaling in cancer has emerged, including added evidence that cancer cells remodel Ca^2+^ signaling through IP_3_Rs to promote migration, tissue invasion, proliferation and survival [54, 55]. Moreover, certain cancers are associated with the up- or down-regulation of specific Ca^2+^ channels or pumps, leading to the proposal that because Ca^2+^ channels, pumps and exchangers are modulated by pharmacological agents, they may be targeted for cancer treatment [56]. Also, recent reports indicate that levels of particular IP_3_R isoforms may serve as potential biomarkers of prognosis or therapeutic response in hematologic malignancies [17, 57].

In conclusion, the findings reported here provide an impetus for targeting Bcl-2-IP_3_R interaction with drugs that mimic the action of BIRD-2. We anticipate that this novel therapeutic strategy will be most effective when combined with ABT-263 or ABT-199, thus inhibiting both of Bcl-2′s anti-apoptotic mechanisms.

## MATERIALS AND METHODS

### Cell culture

Human leukemia, lymphoma, and MM cell lines were cultured in RPMI-1640 media with 10% FBS. Murine fibroblast 3T3 cells and human embryonic kidney HEK293T cells were cultured in DMEM media with 10% FBS. Each medium was supplemented with 100 μM nonessential amino acids, 2 mM L-glutamine, and in MM lines 1 mM sodium pyruvate. Primary human chronic lymphocytic leukemia (CLL) cells were isolated from peripheral blood of adult patients with CLL as previously described [16]. The use of de-identified human subject samples was approved by the institutional review board of Case Western Reserve University School of Medicine.

### Reagents

Hoechst 33342 and Fura-2-AM were purchased from Life Technologies. Z-VAD-FMK was purchased from BIOMOL. ABT-263, ABT-199, and Ibrutinib (all from Selleck) were prepared in DMSO. Peptides were synthesized by GenScript and were prepared in water. BIRD-2 was initially described and referred to as TAT-IDP_DD/AA_ [16] and has the following sequence: RKKRRQRRRGGNVYTEIKCNSLLPLAAIVRV. Two control peptides, neither of which had any detectable activity, nor any differences in effects, were used interchangeably in this study. One is a scrambled form of BIRD-2 that has been referred to previously [16] with sequence: RKKRRQRRRGGDLNEVTCSLIVDRINPVKLY. The other has sequence identical to BIRD-2 except for point mutations designed to inhibit function: RKKRRQRRRGGNVYTEGKCNSGGPLAAIGRV. Peptides were purified by liquid chromatography/mass spectrometry to more than 95% purity, and were quantified by amino acid analysis.

### MTS assay

Cell viability was measured using the MTS assay using the Cell Proliferation Kit (Promega). Adherent cells were plated at 5,000/well in 96-well cell culture plates and incubated overnight before use. Non-adherent cells (leukemia, lymphoma, and myeloma cell lines and primary CLL cells) were suspended at 2 × 10^5^/ml in culture media. All cells were treated with synthetic peptides for time periods indicated in the text, followed by addition of 20 μL MTS reagent to 100 μL cell culture media and incubation for four hours before cell lysis by 1% SDS. Absorbance at 490 nm was measured using a VICTOR^3^ microplate reader (Perkin Elmer). The percentage of viable cells was determined by comparing the absorbance in drug-treated cells to the absorbance in vehicle-treated cells.

### CellTiter-Glo assay

Cells were added to 96-well plates and treated as above with the concentrations of drugs indicated in the text. After 24 hours, CTG assay reagent (Promega) was diluted 5-fold in PBS, and 80 μL of the diluted reagent was added to each well. Plates were agitated for 2 min and then incubated at rest for 10 min. Total luminescence of each well, corresponding to ATP content, was recorded using a VICTOR^3^ microplate reader (Perkin Elmer).

### Hoechst staining to detect apoptotic nuclei

MM: Cells were plated at 2 × 10^6^/mL and treated with vehicle (water), BIRD-2ctrl, or BIRD-2 at the indicated concentrations for the time periods indicated in the figure legends. FL: Cells were treated with ABT-199, BIRD-2 or ABT-199+BIRD-2 or no treatment at the indicated concentrations for 20 hours. Cells were incubated with 10 μg/mL of Hoechst 33342 for 10 minutes and images were taken with a Axiovert S100 (Zeiss) Fluorescence Microscope equipped with a 40 × oil objective (Zeiss) with excitation/emission at 350/535 nm. For each treatment, 200–600 cells were analyzed for presence of chromatin condensation characteristic of apoptosis.

### Immunocytochemistry to detect active Bax

The procedure was described previously [58] with modifications noted here. Briefly, cells were plated at 1×10^6^/mL and treated with 10 μM BIRD-2, 10 μM BIRD-2ctrl, or 2.5 μM staurosporine. After 4 hours of incubation, cells were collected, washed twice with ice-cold PBS, and fixed for 10 minutes at room temperature in 2% paraformaldehyde in PBS. Cells were then washed twice with ice-cold PBS and permeabilized for 30 minutes at room temperature in a solution containing 0.1% Triton X-100 and 2% BSA in PBS. Following two additional washes with ice-cold PBS, samples were incubated for 30 minutes at room temperature in blocking buffer containing 2% BSA in PBS and incubated overnight at 4°C with 0.65 μg Bax 6A7 antibody (BD Pharmingen) or 0.65 μg Normal Mouse IgG antibody (Santa Cruz Biotechnology) in PBS containing 2% BSA. After two washes with PBS, samples were incubated for 1 hour at room temperature in the dark with AlexaFluor 488 Goat Anti-Mouse secondary antibody (Invitrogen) at a concentration of 1:175 in PBS containing 2% BSA. Samples were washed twice in the dark and images were acquired through a QImaging camera on a Nikon Eclipse TE2000-S microscope equipped with a 20× objective using MetaMorph software.

### Calcium imaging

Techniques for single-cell digital imaging of Fura-2-AM-loaded cells were described previously [16, 53].

### Immunoblotting

The procedures were described previously [53]. The following antibodies were used: anti-Bcl-2 (Santa Cruz, sc-7382); anti-Mcl-1 (Biovision, 3035-100); anti-Bim (Cell Signaling, 2933); anti-PARP (Cell Signaling, 9542); anti-cleaved Caspase-3 (Cell Signaling, 9661); and, anti-beta-actin (Sigma, A2228).

### RT-PCR

RNA was isolated from cells treated with either BIRD-2 or BIRD-2ctrl and RT-PCR used to assess the expression levels for Bcl-2 and Bim using previously described methods [59].

### Xenograft model

Animal protocols were approved by the Institutional Animal Care and Use Committee, Case Western Reserve University. Tumor xenografts were established by subcutaneous injection of 9 × 10^6^ NCI-H929 cells into the flank of 5–7 week old NCR nu/nu mice. Tumor size was measured by caliper and tumor volume was estimated by the formula as: Volume (mm^3^) = 0.5 × (length) × (width)^2^, where length and width represents the long and short diameter of a tumor, respectively. When tumors reached an average size of 60–80 mm^3^, peritumoral injection of saline or BIRD-2 (at the dose of 10 mg/kg) was performed every 2-3 days for a total of four injections. Mice were sacrificed 18 days after tumor inoculation and tumors were excised and weighed.

### Statistical analysis

A Mann-Whitney *u* test (xenograft experiments) or Student *t* test (apoptosis and viability assays) was used to determine significant differences between two treatment groups. A two-tailed *p*-value of 0.05 or 0.01 (as indicated in text) was the threshold for significance.
